# Supplementary material for: MetaRibo-Seq measures translation in microbiomes
Source: Nat Commun. 2020 Jun 29;11:3268. doi: 10.1038/s41467-020-17081-z (PMC7324362; doi:10.1038/s41467-020-17081-z)
Supplement: Supplementary file 10 — Supplementary Data 7 [file 41467_2020_17081_MOESM10_ESM.zip › File2/Confidence_High_Taxonomy/241483_out.krona.html]

Javascript must be enabled to view this page.

members
magnitude
magnitudeUnassigned
count
unassigned
taxon
rank

241483\_out

7

2
superkingdom
7

7
phylum
1239

7
class
186801


SRS103987\_contig\_number\_2544
7
186802
1
order

6
186803
family

genus
572511
6

species
33038
6

SRS011405\_contig\_number\_18000SRS016267\_contig\_number\_8094SRS017521\_contig\_number\_16543SRS057717\_contig\_number\_7529SRS077730\_contig\_number\_contig-100\_38841.38841SRS149879\_contig\_number\_contig-100\_35785.35786
